# Day-to-Day Population Movement and the Management of Dengue Epidemics

**DOI:** 10.1007/s11538-016-0209-6

**Published:** 2016-10-04

**Authors:** Jorge A. Falcón-Lezama, Ruth A. Martínez-Vega, Pablo A. Kuri-Morales, José Ramos-Castañeda, Ben Adams

**Affiliations:** 1 Centro de Investigaciones sobre Enfermedades Infecciosas, Instituto Nacional de Salud Publica, Universidad 655, Colonia Sta. Maria Ahuacatitlán, Cerrada Los Pinos y Caminera. C.P., 62100 Cuernavaca, Morelos Mexico; 2 Organizacion Latinoamericana de Fomento a la Investigacion en Salud, Calle 110 No. 21-30, Of. 604, Bucaramanga, Santander Colombia; 3 Subsecretaría de Prevención y Promoción de la Salud, Lieja 7, 1er piso, Colonia Juárez, Del. Cuauhtémoc, C.P. 06600 Ciudad de Mexico, Mexico; 4 UTMB Center for Tropical Diseases, University of Texas Medical Branch, 301 University Blvd., Galveston, TX 77555-0435 USA; 5 Department of Mathematical Sciences, University of Bath, Bath, BA27AY UK; 6 Carlos Slim Health Institute, Lago Zurich 245, Edif. Presa Falcón piso 20, Ampliación Granada. Del. Miguel Hidalgo, C.P. 11529 Ciudad de Mexico, Mexico

**Keywords:** Epidemic model, Metapopulation, Host–vector, Control, Demography

## Abstract

Dengue is a growing public health problem in tropical and subtropical cities. It is transmitted by mosquitoes, and the main strategy for epidemic prevention and control is insecticide fumigation. Effective management is, however, proving elusive. People’s day-to-day movement about the city is believed to be an important factor in the epidemiological dynamics. We use a simple model to examine the fundamental roles of broad demographic and spatial structures in epidemic initiation, growth and control. We show that the key factors are local dilution, characterised by the vector–host ratio, and spatial connectivity, characterised by the extent of habitually variable movement patterns. Epidemic risk in the population is driven by the demographic groups that frequent the areas with the highest vector–host ratio, even if they only spend some of their time there. Synchronisation of epidemic trajectories in different demographic groups is governed by the vector–host ratios to which they are exposed and the strength of connectivity. Strategies for epidemic prevention and management may be made more effective if they take into account the fluctuating landscape of transmission intensity associated with spatial heterogeneity in the vector–host ratio and people’s day-to-day movement patterns.

**Electronic supplementary material:**

The online version of this article (doi:10.1007/s11538-016-0209-6) contains supplementary material, which is available to authorized users.

## Introduction

Dengue fever is the most important mosquito-borne viral disease in the world. It is caused by infection with any of the four serotypes of dengue virus. Nearly half of the human population lives in a dengue transmission area. A number of vaccines are under development, including the tetravalent Dengvaxia (CYD-TDV) which has been approved for use in several countries and recommended for introduction in areas with high endemicity (WHO 2016). Until the efficacy of this vaccine is properly established, however, control measures will continue to rely on the reduction in transmission between people and mosquitoes. As an arthropod-borne virus, the basic process of dengue transmission is well understood. An infected vector transmits the virus to a susceptible host who, after an intrinsic incubation period, transmits the virus to another vector which, following an extrinsic incubation period, starts a new cycle. This process is, however, influenced by numerous factors including climate variation, human migration, immune cross-reaction, vertical transmission and widespread asymptomatic, but transmissible, infection (Adams et al. 2006; Kyle and Harris 2008; Adams and Boots 2010; Descloux et al. 2012; Yoon et al. 2012; Rabaa et al. 2013). At the scale of a city or conurbation the pattern of people’s local, day-to-day movements is emerging as a key factor shaping dengue epidemics. Here we develop a mathematical model to improve our understanding of how quotidian urban movement behaviour contributes to the risk of dengue epidemics, and the epidemic trajectories when they do occur, and consider transmission control strategies that account for these patterns.

Human mobility is known to be an important factor in infectious diseases epidemics (Prothero 1977; Peiris et al. 2003; Merler and Ajelli 2010). The complexity of human mobility is a challenge for field studies, but it has been estimated and characterised by several methods including census surveys (Weber et al. 2003), cell phone usage (Wesolowski et al. 2012) and GPS tracking (Seto et al. 2007). Recent studies have started to examine how human mobility patterns are related to dengue transmission (Vazquez-Prokopec et al. 2009, 2013; Stoddard et al. 2009). Understanding this relationship is particularly important because current dengue control measures rely on the management of mosquito vectors or the interruption of human–mosquito contact. The World Health Organisation recommends that these control activities target residential properties, their immediate vicinity and other locations where human–vector contact occurs (WHO 2009). In practice, these activities tend to be focused on residential properties due to limitations on cost, delivery and sustainability (Chang et al. 2011). In addition, there is a lack of reliable information about human–vector contact outside of peridomestic areas. Recent field studies have reported the possibility that such areas are important for dengue transmission in Southeast Asia and in South America (Porter et al. 2005; Stoddard et al. 2013) but the extent of their role has yet to be clarified.

Complementary to field studies, mathematical models have provided important insights into the role of city-scale human mobility in dengue transmission. Data-driven individual-based simulation models incorporate detailed movement behaviours as a matter of course, although analysis of these models does not usually focus on the specific role of the mobility component (e.g. Chao et al. 2012; Karl et al. 2014). Some more conceptual models have specifically focussed on the role of the mobility component. The simplest models include random movement (Pongsumpun et al. 2008; De Castro Medeiros et al. 2011) or regular commuter movement between two locations (Barmak et al. 2014; Nevai and Soewono 2013). More complex models include multiple locations (Adams and Kapan 2009) and multiple population groups (Cosner et al. 2009; Iggidr et al. 2014; Xiao and Zou 2014; Bichara and Castillo-Chavez 2015). Conceptual frameworks for modelling movement can be broadly categorised as Eulerian or Lagrangian. Eulerian approaches observe the flow of individuals through fixed locations; individuals are labelled with their current location but not with additional identifiers such as their origin or residence. This method works well for models of random or migratory movement. Lagrangian approaches track individuals’ movements through all locations; individuals are labelled with their current location and identifiers such as their origin or residential location. This method works well for models of commuter movement (Okubo and Levin 2001; Cosner et al. 2009; Bichara and Castillo-Chavez 2015).

The study we report here is motivated by the observation that, in broad terms, the daily movement patterns of a population may be a combination of commuter movement and random movement, often associated with different demographic groups. We explore this idea by developing a fairly simple multi-patch multi-group model framework that accounts for heterogeneous movement patterns and diurnal population structures. This approach means that our model should not be viewed in direct comparison with detailed individual-based simulation models. Instead it offers a complementary perspective, based on the abstraction of motifs which may occur many times within more detailed models, that is sufficiently simple and transparent to elucidate the underlying mechanisms driving the epidemiological dynamics. We show how the epidemic risk in the whole population is made up of contributions from different demographic groups with exposure to different mosquito populations. We show how the epidemic trajectory through each of these groups is influenced by the mosquito populations in the places they visit, and the other groups that visit those places. Finally, we consider how those factors affect epidemic management and prevention strategies.

## Mathematical Model

We formulate the model as a deterministic compartmental system expressed in terms of ordinary differential equations. In addition to employing analytic and numerical methods to study this system, we simulate it stochastically by re-formulating it as an agent-based model. The model can be thought of as being composed of two submodels. One corresponds to the spatial and demographic population structure, the other to the epidemiological dynamics.

### Population Structure and Movement Submodel

**Fig. 1:**
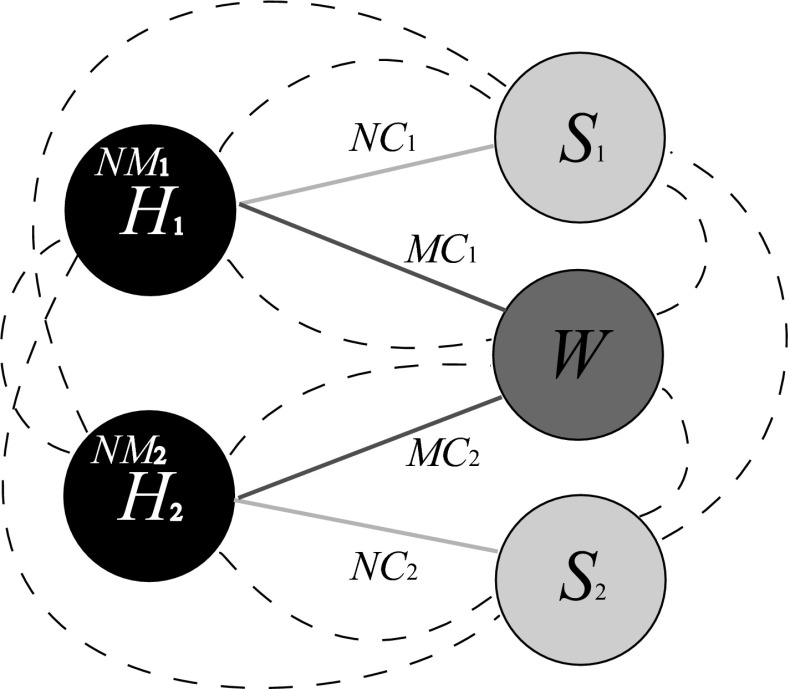
Schematic diagram of spatial structure and population movement. $H_1$ and $H_2$ are residential patches, and non-mobile groups ($NM_1, NM_2$) remain here all the time. $S_1$ and $S_2$ receive non-mixing commuters ($NC_1, NC_2$) from $H_1$ or $H_2$, but not both. *W* receives mixing commuters ($MC_1, MC_2$) from $H_1$ and $H_2$. The highly mobile population (*HM*) moves between all patches at random, as indicated by the *dashed lines*

The spatial environment is divided into five patches (Fig. 1). Two patches ($H_i, i = 1, 2$) represent geographically distinct residential areas. Two patches ($S_i, i = 1, 2$) represent locations that are geographically distinct from the residential areas but tightly coupled socially so that the majority of people that commute to patch $S_i$ are resident in patch $H_i$. These patches might, for instance, be schools. Note that we will sometimes refer to the *H*, or *S*, patches by which we mean $H_1$ and $H_2$, or $S_1$ and $S_2$. The fifth patch (*W*) represents a location which receives commuters from both residential areas. This patch might, for instance, be a large, centralised workplace. This five-patch formulation was considered the minimum necessary to provide an insight into the complex interplay of spatial and temporal structures. The population moves between the patches. It is divided into seven groups based on movement behaviour. Groups $NM_i, (i = 1, 2)$ are non-mobile. They remain in their designated residential patch ($H_i$) at all times. These groups may be broadly interpreted as the very old and the very young. Groups $NC_i, (i = 1, 2)$ are non-mixing commuters. They commute to and from a residential patch ($H_i$) and the corresponding uniquely coupled destination patch ($S_i$). These groups may be broadly interpreted as school children. Groups $MC_i, (i = 1, 2)$ are mixing commuters. They commute to and from a residential patch ($H_i$) and the shared destination patch (*W*). These groups may be broadly interpreted as the regular workforce of large centres such as offices, factories, hospitals or markets. Note that we will sometimes refer to the *NM* group by which we mean $NM_1 + NM_2$, and similarly for *NC* and *MC* groups. Group *HM* is highly mobile. They move between all five patches frequently and at random. This group may be broadly interpreted as peripatetic workers such as taxi drivers, small tradesmen or salesmen. The proportion of the total population in group *X* is denoted $p_X$, and the total size of group *X* in patch *Y* is denoted $N_X^Y$. Individuals in the $NC_i$ and $MC_i$ groups leave their residential patch at rate $\rho _{NC}, \rho _{MC}$ and return at rate $\tau _{NC}, \tau _{MC}$. Individuals in the *HM* group move to a random new patch at rate $\rho _{HM}$.

### Epidemic Submodel

Each population group is subdivided into compartments according to disease state: susceptible (*S*), exposed but not yet infectious (*E*), infectious (*I*), recovered and immune (*R*). The total size of the population of group *X* in patch *Y* and in disease state *Z* is denoted $Z_X^Y$. Summing over all patches gives the total size of the population in group *X* and state $Z, Z_X = \sum _Y Z_X^Y$. Each patch *Y* also has a mosquito population of size $N_V^Y$. Each mosquito population is subdivided into compartments according to disease state: susceptible (*S*), exposed but not yet infectious (*E*) and infectious (*I*). The total size of the mosquito population in patch *Y* in state *Z* is denoted $Z_V^Y$. Mosquitoes do not move between patches. The mosquito population in each patch has a constant mortality rate $\mu$, and birth rate $\frac{\kappa N}{5}\mu$ where *N* is the number of people in the entire system. So, at equilibrium, each patch has one-fifth of the mosquito population and the vector–host ratio in the whole system is $\kappa$. It would be straightforward to modify the mosquito birth rates such that the vector–host ratio in the whole system is still $\kappa$ but mosquito population sizes are heterogeneous between patches. Non-uniform mosquito distributions are likely typical in the real world. However, we maintain a uniform distribution in order to focus on the role of human movement. We return to this point in the discussion.

Mosquitoes are assumed to bite at a constant rate $\beta$ regardless of the size of the human population in their patch. Consequently, susceptible people in group *X* and patch *Y* are infected at rate $\displaystyle \beta \frac{S_X^Y}{N_\varSigma ^Y} I_V^Y$ where $N_\varSigma ^Y$ is the total number of people in patch *Y*. Following infection, people enter the exposed state and progress to the infectious state at rate $\varepsilon$. They recover from the infectious state at rate $\gamma$ and then retain lifelong immunity. So, if the recovered population is initially zero, $\sum _X R_X(0) = 0$, then $\sum _X R_X(t)$ is the total number of people that have been infected and recovered at time *t*, a measure of the epidemic size. Susceptible mosquitoes in patch *Y* are infected at rate $\displaystyle \beta \frac{I_\varSigma ^Y}{N_\varSigma ^Y}S_V^Y$ where $I_\varSigma ^Y$ is the total number of infected people in patch *Y*. Following infection, mosquitoes enter the exposed state and progress to the infectious state at rate $\varepsilon _V$. They do not recover. The complete system is composed of 72 equations. The part of the system representing the people in the non-mixing commuter ($NC_i$) groups is given in Eqs. (1–8). The equations for the mixing commuter ($MC_i$) groups are similar. The equations for the non-mobile ($NM_i$) groups are similar, except there are no movement terms, and these groups are restricted to the residential patches. The equations for the highly mobile group (*HM*) are similar except there is a set of equations for each of the five patches, the rate at which the population in state *Z* leaves patch *Y* is $\rho _{HM}Z_{HM}^Y$ and the rate at which the population arrives from each of the other four patches $\hat{Y}$ is $\frac{\rho _{HM}}{4} Z_{HM}^{\hat{Y}}$. All of these equations are provided in full in the Appendix. The part of the system representing the mosquitoes in patch *Y* is given by Eqs. (9–11).

Similar multi-patch multi-host frameworks have been used in other recent studies. Iggidr et al. (2014) consider a model with *n* groups, which may be thought of as locations, and generalised terms for cross-transmission between these groups. Bichara and Castillo-Chavez (2015) consider a model with multiple locations and multiple socio-demographic host groups distinguished by the expected proportion of time spent in each location (the residence time). Both of these studies are based on quite general Lagrangian models and focus on rigorous mathematical analysis of the existence and stability of equilibrium states.

#### Equations for Non-mixing Commuter Groups and Mosquitoes

1 $$\begin{aligned} \frac{\mathrm{d}S_{NC_i}^{H_i}}{\mathrm{d}t}= & {} -\beta I_V^{H_i}\frac{S_{NC_i}^{H_i}}{N_\varSigma ^{H_i}} - \tau _{NC}S_{NC_i}^{H_i} + \rho _{NC}S_{NC_i}^{S_i} \end{aligned}$$

2 $$\begin{aligned} \frac{\mathrm{d}E_{NC_i}^{H_i}}{\mathrm{d}t}= & {} \beta I_V^{H_i}\frac{S_{NC_i}^{H_i}}{N_\varSigma ^{H_i}} - \varepsilon E_{NC_i}^{H_i} - \tau _{NC}E_{NC_i}^{H_i} + \rho _{NC}E_{NC_i}^{S_i}\end{aligned}$$

3 $$\begin{aligned} \frac{\mathrm{d}I_{NC_i}^{H_i}}{\mathrm{d}t}= & {} \varepsilon E_{NC_i}^{H_i} - \gamma I_{NC_i}^{H_i} - \tau _{NC}I_{NC_i}^{H_i} + \rho _{NC}I_{NC_i}^{S_i}\end{aligned}$$

4 $$\begin{aligned} \frac{\mathrm{d}R_{NC_i}^{H_i}}{\mathrm{d}t}= & {} \gamma I_{NC_i}^{H_i} - \tau _{NC}R_{NC_i}^{H_i} + \rho _{NC}R_{NC_i}^{S_i} \end{aligned}$$

5 $$\begin{aligned} \frac{\mathrm{d}S_{NC_i}^{S_i}}{\mathrm{d}t}= & {} -\beta I_V^{S_i}\frac{S_{NC_i}^{S_i}}{N_\varSigma ^{S_i}} - \rho _{NC}S_{NC_i}^{S_i} + \tau _{NC}S_{NC_i}^{H_i} \end{aligned}$$

6 $$\begin{aligned} \frac{\mathrm{d}E_{NC_i}^{S_i}}{\mathrm{d}t}= & {} \beta I_V^{S_i}\frac{S_{NC_i}^{S_i}}{N_\varSigma ^{S_i}} - \varepsilon E_{NC_i}^{S_i} - \rho _{NC}E_{NC_i}^{S_i} + \tau _{NC}E_{NC_i}^{H_i}\end{aligned}$$

7 $$\begin{aligned} \frac{\mathrm{d}I_{NC_i}^{S_i}}{\mathrm{d}t}= & {} \varepsilon E_{NC_i}^{S_i} - \gamma I_{NC_i}^{S_i} - \rho _{NC}I_{NC_i}^{S_i} + \tau _{NC}I_{NC_i}^{H_i}\end{aligned}$$

8 $$\begin{aligned} \frac{\mathrm{d}R_{NC_i}^{S_i}}{\mathrm{d}t}= & {} \gamma I_{NC_i}^{S_i} - \rho _{NC}R_{NC_i}^{S_i} + \tau _{NC}R_{NC_i}^{H_i} \end{aligned}$$

**Table 1:** Parameter definitions and values used in all analysis and simulations in this paper unless otherwise stated. All rates are per day

Parameter	Meaning	Value
*N*	Total human population size	4000
$p_{NC}$	Non-mixing commuter proportion, evenly distributed between groups 1 & 2	0.25
$p_{MC}$	Mixing commuter proportion, evenly distributed between groups 1 & 2	0.3
$p_{HM}$	Highly mobile proportion	0.05
$p_{NM}$	Non-mobile proportion, evenly distributed between groups 1 & 2	0.4
$\rho _{NC}$	Rate at which non-mixing commuters move from *H*-patch to *S*-patch	1.5
$\rho _{MC}$	Rate at which mixing commuters move from *H*-patch to *W*-patch	1.5
$\rho _{HM}$	Rate at which highly mobile individuals leave current patch	8
$\tau _{NC}$	Rate at which non-mixing commuters move from *S*-patch to *H*-patch	3
$\tau _ {MC}$	Rate at which mixing commuters move from *W*-patch to *H*-patch	3
$\varepsilon _h$	Incubation rate in people	0.25
$\gamma$	Recovery rate of people	0.2
$\kappa$	Overall vector–host ratio	1
$\varepsilon _V$	Incubation rate in mosquitoes	0.14
$\mu$	Mortality rate in mosquitoes	0.14
$\beta$	Transmissible biting rate of mosquitoes	0.26

where $i = 1$ or 2 and $N_\varSigma ^{H_i}$ is the total human population in patch $H_i$.

9 $$\begin{aligned} \frac{\mathrm{d}S_{V}^{Y}}{\mathrm{d}t}= & {} \frac{\kappa N}{5}\mu - \beta S_V^Y\frac{I_\varSigma ^Y}{N_\varSigma ^Y} - \mu S_V^Y \end{aligned}$$

10 $$\begin{aligned} \frac{\mathrm{d}E_{V}^{Y}}{\mathrm{d}t}= & {} \beta S_V^Y\frac{I_\varSigma ^Y}{N_\varSigma ^Y} - \varepsilon _V E_V^Y - \mu E_V^Y \end{aligned}$$

11 $$\begin{aligned} \frac{\mathrm{d}I_{V}^{Y}}{\mathrm{d}t}= & {} \varepsilon _V E_V^Y - \mu I_V^Y. \end{aligned}$$

### Parameterisation

The parameter values used for computational analysis of the model are given in Table 1. The epidemiological parameters are reasonable estimates for dengue. $\varepsilon _h = 0.25$ corresponds to an expected incubation period in people of 4 days (Rudolph et al. 2014; Snow et al. 2014). $\gamma = 0.2$ corresponds to an expected infectious duration in people of 5 days (Gubler et al. 1981; Duong et al. 2015). $\varepsilon _v = 0.14$ corresponds to an expected incubation period in mosquitoes of 7 days; in reality, the incubation period is strongly dependent on temperature, but 7 days is plausible for many areas (Rohani et al. 2009). $\mu = 0.14$ corresponds to a mosquito life expectancy of 7 days; female Aedes mosquitoes are reported to live up to 30 days in laboratory conditions (Xavier et al. 1991), but the greatly increased hazards of natural conditions make 7 days a reasonable estimate. The transmissible biting rate of 0.26 corresponds to approximately one bite every 4 days. Female Aedes mosquitoes require blood in order to complete their gonotrophic cycle. According to Wong et al. (2011), the length of the gonotrophic cycle is 3–4 days. The vector–host ratio $\kappa$ gives a person–person basic reproduction number $R_0^2$ of approximately 2, a reasonable value for dengue (Kuno 1997). The total human population size *N* can be scaled out of the ordinary differential equation model, and so its value is arbitrary. However, it is fundamental in the agent-based model and *N* = 4000 was the largest number for which simulations could be computed sufficiently quickly. The other demographic parameters are broad estimates based on cohort studies conducted by our group in Morelos, Mexico (Martínez-Vega et al. 2015; Falcón-Lezama et al. submitted). The proportions of people in each group $p_{NM} = 0.4, p_{NC} = 0.25, p_{MC} + p_{HM} = 0.35$ and movement rates are based on age distribution and activity type data. The movement rates $\rho _X, \tau _X$ determine the expected proportion of time individuals in each group spend in each patch. For commuting groups, the proportion of time spent away from the residential location is $\rho _X/(\rho _X + \tau _X) = 1/3$. For individuals in the highly mobile group, the expected sojourn time in each patch is 1/8 day, representative of frequent movement but with visits long enough for interaction with the local mosquito population.

## Results

The model population is structured into two non-mixing ‘local’ subpopulations, each composed of non-mobile ($NM_i$) residents in $H_i$ and non-mixing commuters ($NC_i$) moving between $H_i$ and $S_i$. The subpopulations are connected by mixing commuters $(MC_i)$ moving between $H_i$ and *W*, and the highly mobile (*HM*) population. We now examine the role of each of these demographic groups in determining key epidemiological characteristics such as the basic reproduction number, the final epidemic size and the maximum prevalence. We particularly focus on the contrasting roles of the two ‘connecting’ groups, the mixing commuters and the highly mobile population. To this end, the total population size is held constant, and the proportion of the population in the *MC* and *HM* groups combined is held constant at $p = p_{MC} + p_{HM}$. However, the distribution of the population between these two groups is adjusted with a parameter *q* where the proportion of the population in the *HM* group is *pq* and the proportion in the two *MC* groups is $p(1 - q)$, divided equally between each of them. If $q = 0$, there are no highly mobile individuals. If $q = 1$, there are no mixing commuters. The number of mosquitoes in each patch is held constant. Consequently, adjusting the population distribution between the *MC* and *HM* groups has two effects. It changes the nature of the connection, and so the dispersal of infection, between the two ‘local’ populations. It also changes the size of the human population present in each patch. This is important because of the dilution effect (Schmidt and Ostfeld 2001). If the size of the human population increases, but the size of the mosquito population remains constant, then frequency-dependent biting means that each person is bitten less often. Hence, the expected number of mosquitoes infected by each infectious person decreases. But the expected number of people infected by each infectious mosquito does not change.

### Basic Reproduction Number

The basic reproduction number $R_0$ is most generally defined as the expected number of secondary infections resulting from a ‘typical’ infected individual in an otherwise susceptible population. It is a measure of the epidemic risk in a disease-free population. In this definition, the so-called typical infected individual is composed of contributions from each infectious population group, both hosts and vectors, in proportion to their representation in the initial exponential growth phase of the epidemic (Diekmann et al. 2013). In the analysis of host–vector models, it is conventional to consider the basic reproduction number over two infection generations, $R_0^2$. This form has the intuitive interpretation of the expected number of people infected by one typical infection person, or the expected number of mosquitoes infected by one typical infected mosquito.

The basic reproduction number is easily found using the next-generation matrix approach. Full details of this method can be found elsewhere (e.g. Diekmann et al. 2010). Briefly, we limit our attention to the infection subsystem, i.e. the equations for those state variables that represent infected states, in our case $E_v^Y, E_X^Y$ and $I_v^Y, I_X^Y$ for all groups *X* and patches *Y*. We linearise this system about the disease-free equilibrium and write the right-hand side of the differential equations as sum of two matrices, *T* which represents transmission, i.e. the production of new infections, and $\varSigma$ which represents transition, i.e. change from one infected state into another. Then, $K_L = -T\varSigma ^{-1}$ is referred to as the Next Generation of Large Domain (Diekmann et al. 2010), or sometimes just the next-generation matrix (Van den Driessche and Watmough 2002). The next-generation matrix of Large Domain can be reduced in dimension if not all infected states are ‘states-at-infection’. These are the states individuals enter immediately following infection, in our case $E_v^Y, E_X^Y$. This reduction is achieved by constructing an auxiliary matrix *E* (as detailed in Diekmann et al. (2010)) such that $K = EK_LE^{-1}$ has dimension equal to the number of states-at-infection and is the next-generation matrix as defined in Diekmann et al. (1990). Element $k_{ij}$ of *K* is the expected number of infections with state-at-infection *i* generated by one individual that becomes infected with state-at-infection *j*. The basic reproduction number $R_0$ is the largest eigenvalue of *K*, which is also the largest eigenvalue of $K_L$.

In the initial exponential growth phase of the epidemic, the relative proportions of each state-at-infection remain constant and can be found from the next-generation matrix *K*. If the host states-at-infection are indexed $1,\ldots ,m$ and the vector states-at-infection are indexed $m+1,\ldots ,n$ then the next-generation matrix has the form $$K = \left( \begin{array}{cc} 0 &{} K_{vh} \\ K_{hv} &{} 0 \end{array}\right) $$ where the matrix $K_{vh}$ encapsulates the infection of hosts by vectors, and $K_{hv}$ encapsulates the infection of vectors by hosts. The two generation matrix is $$K^2 = \left( \begin{array}{cc} K_{hh} &{} 0 \\ 0 &{} K_{vv} \end{array} \right) $$ where the elements of $K_{hh} = K_{vh}K_{hv}$ are the expected number of host infections with state-at-infection *i* from one infected host with state-at-infection *j*, and similarly with respect to vectors for $K_{vv} = K_{vh}K_{hv}$. The dominant eigenvalue of $K^2$ is $R_0^2$, which is also the dominant eigenvalue of both $K_{hh}$ and $K_{vv}$. The eigenvector $\mathbf {w}$ associated with $R_0^2$ has the form $$\mathbf {w} = \left( \begin{array}{cc}\mathbf {h} \\ \mathbf {v} \end{array}\right) $$ where $\mathbf {h}$ is an eigenvector associated with $R_0^2$ for $K_{hh}$, and $\mathbf {v}$ is an eigenvector associated with $R_0^2$ for $K_{vv}$ (Turner et al. 2013). Normalising $\mathbf {h}$ such that the sum of the elements $\sum _{j=1}^m h_j = 1$ gives the proportional representation of each host state-at-infection in the initial epidemic growth phase, i.e. the composition of the ‘typical’ infected host.

The sum of column $j = 1,\ldots ,m$ of $K_{hh}^2$ is the expected number of host infections resulting (via vector-mediated transmission) from one infected host with state-at-infection *j*. We label this $R_{0j}^2$. The contribution of each host state-at-infection *j* to $R_0^2$ is given by $h_jR_{0j}^2$, the proportion of the infectious population in that state, and its potential for onward transmission. To see this, let $\mathbf {1}$ be the column vector of length *m* with all entries 1. Then $\mathbf{1}^T K_{hh}$ is a row vector of length *m* with elements $R_{0j}^2$. The expected number of infections from a typical infected host in the initial epidemic growth phase is $\sum _{j = 1}^m h_jR^2_{0j} = \mathbf{1}^T K_{hh}{} \mathbf{h} = \mathbf{1}^T R_0^2 \mathbf{h} = R_0^2\sum _{j=1}^m h_j = R_0^2$. We find the total contribution to $R_0^2$ of hosts in group *X*, across all patches, by summing $h_jR_{0j}^2$ over all states *j* that make up group *X*. In addition, we find $P_X$, the proportion of hosts in group *X*, across all patches, during the initial epidemic growth phase by summing the appropriate $h_j$ terms.

**Fig. 2:**
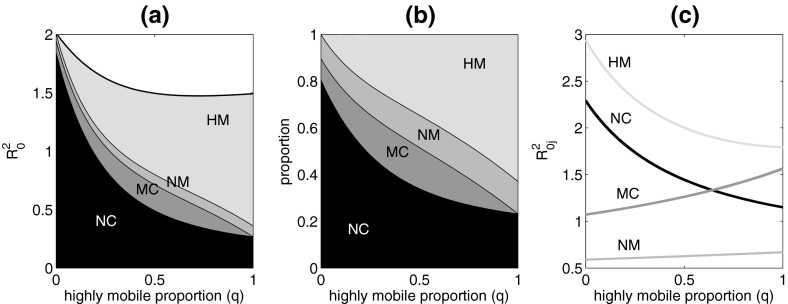
The basic reproduction number and its constituents as a function of the proportion *q* of the total highly mobile (*HM*) and mixing commuter (*MC*) population that is in the *HM* group. **a** Basic reproduction number $R_0^2$ for the entire metapopulation. Layers show the contribution to $R_0^2$ from each demographic group $X = NM, NC, MC, HM$. **b** Proportion $P_X$ of infections during the initial phase of the epidemic that occur in each demographic group $X = NM, NC, MC,HM$. **c** Reproduction numbers $R_{0j}^2$ for states-at-infection *j*—the expected number of people infected from one infected person with state-at-infection *j* during the initial phase of the epidemic. States-at-infection *j* are $E_X^Y$ for patch *Y* and group *X*. However, in this figure, for each group $X = NM, NC, MC, HM$ the reproduction numbers are almost identical for all patches *Y* in which that group occurs. This means that the number of infections caused by an individual in any given group *X* is only very weakly dependent on the patch *Y* in which that individual was infected. So we label the lines in the graph by group *X*, but not patch *Y*. All parameters as in Table 1

Figure 2 shows how the value and composition of the basic reproduction number depend on the population distribution *q* between the *MC* (mixing commuter) and *HM* (highly mobile) groups. The components of this figure were computed numerically from the next-generation matrix *K*, using the column sums, dominant eigenvalue and associated eigenvector as described above. As *q* increases, and the *HM* group becomes larger, $R^2_0$ decreases, saturating when the *HM*–*MC* ratio is about 1:1 ($q = 0.5$). When $q = 0$, about 80 % of infections in the initial phase of the epidemic occur in the *NC* (non-mixing commuter) group. When the *HM*–*MC* ratio is low, the number of people in the *H* and *W* patches at any time is large compared to the *S* patches. So dilution means that there is relatively little transmission in the *H* and *W* patches. As the *HM*–*MC* ratio increases, individuals in the mixing commuter group become highly mobile and disperse among all the patches. There is a net increase in the number of people in the *S* patches, diluting transmission. There is a small net decrease in the *H* patches, and a large net decrease in the *W* patch, both increasing transmission. Consequently the contribution of the *NC* group to $R^2_0$ decreases. The contribution of the *NM* groups increases slightly but, because all groups spend some time in the *H* patches, there is strong dilution and transmission in these patches is never a major component of $R^2_0$.

The contribution of the *MC* group to $R^2_0$ initially increases as the *HM*–*MC* ratio increases. The decrease in the local population size intensifies transmission in the *W* patch. However, as the *HM*–*MC* ratio increases further, the declining size of the *MC* group reduces its contribution to $R^2_0$, despite the transmission intensification. The contribution of the *HM* group increases because this group becomes larger. Furthermore, *HM* individuals are exposed, for at least some of the time, to all of the mosquito subpopulations including those in patches where the transmission intensity is high due to the small number of people present. When $q = 1$, about 25 % of infections in the initial phase of the epidemic occur in the *NC* group, and about 60 % occur in the *HM* group.

### Epidemic Trajectory

We now consider numerical solutions of the ordinary differential equation system to examine how the demographic structure affects the transient epidemiological dynamics beyond the initial epidemic phase. Stochastic simulations with the agent-based model show broadly consistent behaviour (Supplementary Figures S1 and S2). Dilution remains an important factor, but the manner in which people spread the infection among distinct mosquito populations also has a role.

**Fig. 3:**
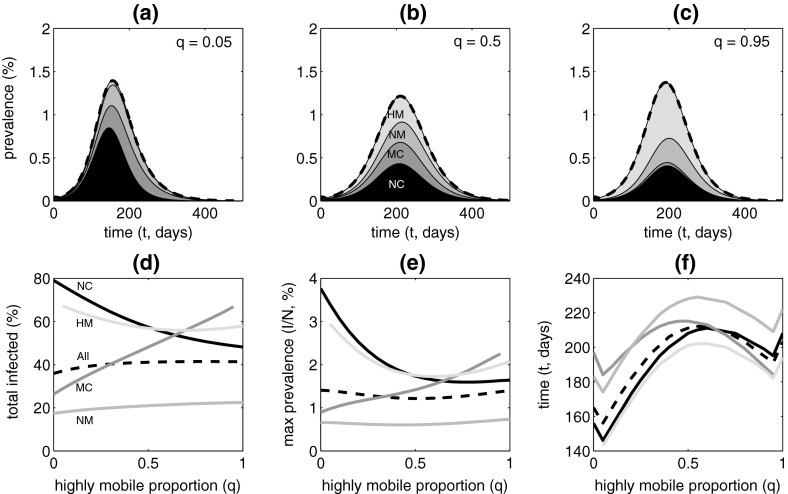
The epidemic trajectory and its constituents as a function of the proportion *q* of the total highly mobile (*HM*) and mixing commuter (*MC*) population that is in the *HM* group. **a**–**c** Prevalence over time as a proportion of the total population ($\sum _X I_X(t)/N$) for $q = 0.05, 0.5, 0.95$. Layers show the contribution from each demographic group ($I_X(t)/N)$. **d** Proportion of the total population ($\sum _X R_X(1000)/N)$, and of each group ($R_X(1000)/N_X$), infected over the entire course of the epidemic. **e** Maximum prevalence in the total population, and in each group, during the epidemic. **f** Time of maximum prevalence in total population and each group. Parameters as in Table 1. Initially, all population groups were entirely susceptible except for five exposed individuals distributed evenly over all 15 exposed classes of the human population, i.e. $S_X^Y(0) = N_X^Y(0) - 5/15, E_X^Y(0) = 5/15$ for all 15 valid combinations of group *X* and patch *Y*

Figure 3a–c shows how prevalence changes over time following the introduction of infection into a susceptible population. When the highly mobile to mixing commuter (*HM*–*MC*) ratio is low, the initial epidemic is driven by the non-mixing commuter (*NC*) group, due to infection in the *S* patches. The infection is slow to spread to groups associated with the *H* and *W* patches due to strong dilution in those patches, and weak connectivity. In particular, the main route by which infection disperses from the mosquito population in the *S* patches to that in the *W* patch is via the *H* patches, where dilution is strongest and weak transmission slows the spread. The infection does, however, become established in these patches eventually and, as the epidemic reaches its peak and declines, the non-mobile (*NM*) and mixing commuter (*MC*) populations account for a large proportion of infections. As the *HM*–*MC* ratio increases, infections become distributed more evenly in proportion to the size of each group throughout the entire epidemic. Although dilution continues to modulate the local transmission intensity in each patch, highly mobile individuals disperse infection from patches where the transmission intensity is high, because few people are present, to patches where the transmission intensity is lower. This dispersal tends to synchronise the epidemics in each population group.

Figure 3d–e shows how key characteristics of the epidemic vary with the *HM*–*MC* ratio. As this ratio increases, there is a marked decrease in the proportion of the *NC* group that is infected. There is a smaller, but notable, decrease in the infected proportion of the *HM* group, although the total size of this group increases. There is a marked increase in the infected proportion of the *MC* group, although the total size of this group deceases, and a slight increase in the infected proportion of the *NM* group. The net impact on the entire population is just a slight increase in the total proportion that is infected. The changes are mainly related to the extent of dilution in the patches people in each group frequent.

As the *HM*–*MC* ratio increases, the maximum prevalence, at the epidemic peak, decreases markedly in the *N*C group, decreases and then increases in the *HM* group, increases markedly in the *MC* group and remains almost constant in the *NM* group. Overall, the maximum prevalence shows only slight variation, reaching a minimum when the *HM*–*MC* ratio is 1:1. When the *HM*–*MC* ratio is low, the maximum prevalence is reached in the *NC* and *HM* groups at about the same time, before the other groups. The local epidemics in the *NC* groups drive the epidemic in the *HM* group, but it is slow to spread to the other groups due to weak connectivity and dilution. When the *HM*–*MC* ratio is intermediate, the maximum prevalence is reached at about the same time in the *NC*, *MC* and *HM* groups. The transmission intensity is similar in the *S* and *W* patches, and they are strongly connected by the *HM* population. When the *HM*–*MC* ratio is high, the maximum prevalence is reached in the *MC* and *HM* groups at about the same time, before the other groups. The local epidemic in the *MC* group drives the epidemic in the *HM* group and builds slightly more slowly in the other groups due to dilution.

## Epidemic Management and Prevention

**Fig. 4:**
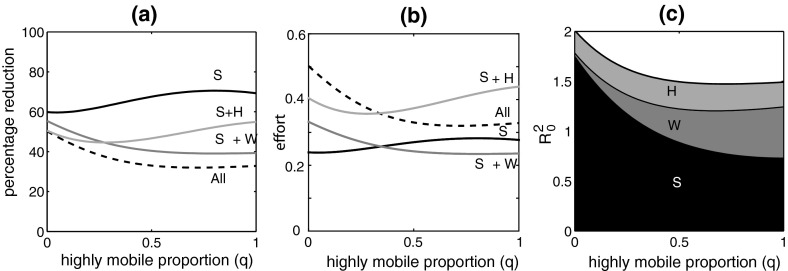
**a** The percentage reduction in different mosquito populations required to achieve $R_0^2 = 1$ as a function of the proportion *q* of the total highly mobile (*HM*) and mixing commuter (*MC*) population that is in the *HM* group. *Black*: reduction in mosquito populations in patches $S_1$ and $S_2$ only. *Dark grey*: reduction in mosquito population in patches $S_1, S_2$ and *W*. *Pale grey*: reduction in mosquito population in patches $S_1, S_2, H_1$ and $H_2$. *Dashed line*: reduction in mosquito population in patches $S_1, S_2, W, H_1$ and $H_2$. **b** The control effort associated with each strategy, found by multiplying the effort in a) by the proportion of patches in which it is applied. **c** The basic reproduction number as a function of *q* with layers showing the contribution to $R_0^2$ from each patch type. Parameters as in Table 1

One of the primary methods of dengue control is fumigation to reduce the size of the adult mosquito population. We consider two aspects, epidemic prevention and epidemic management. A disease-free population will remain disease-free if the basic reproduction number is less than 1. Our model parameterised as in Table 1 has $R_0^2 > 1$ for all values of *q*. So, for epidemic prevention, we consider the extent to which the mosquito population in each patch must be reduced in order to reach the threshold $R_0^2 = 1$. Figure 4c shows the contribution of each mosquito population to $R_0^2$. Figure 4a shows the reduction in each of these populations required for $R_0^2 = 1$. When the *HM*–*MC* ratio is low, epidemic prevention can be achieved by reducing the mosquito population by 60 % in the *S* patches, or by 55 % in the *S* and *W* patches, or by just over 50 % in the *S* and *H* patches, or by 50 % in all of the *S*, *H* and *W* patches. Targeting the *W* or *H* patches alone cannot prevent an epidemic.

We evaluate the control effort associated with each of these strategies by assuming it is proportional to the total reduction in the mosquito population size. So, given equal mosquito population sizes in each patch, the effort required for a reduction by a proportion *r* in *n* of the five patches is $\frac{n}{5} r$. Figure 4b shows the effort required by each strategy. For *q* close to 0, it is most efficient to target the *S* patches only. As *q* increases, it becomes beneficial to include the *W* patch because of high transmission associated with the localised increase in the vector–host ratio in that patch. Strategies that include the *H* patches are less efficient because low vector–host ratios in these patches mean that the extra effort of treating two additional areas has a limited return. For larger values of *q*, which lead to high vector–host ratios in the *W* patch, it may be more efficient to target all patches than just the *S* and *H* patches. As the *HM*–*MC* ratio increases, epidemic prevention requires a greater reduction in the mosquito population if only the *S* patches are targeted. The benefit of including the *H* patches in the prevention strategy remains similar, but the advantage of including the *W* patch increases markedly. The focus here is on the role of the mosquito population in each patch, not the human population groups, although it is the latter that are usually observed in epidemiological data. In this particular model system, an epidemic requires transmission, i.e. the presence of mosquitoes, in the *S* patches. Hence, epidemic prevention requires reducing the mosquito populations in these patches. However, as the *HM*–*MC* ratio increases, the role of the *S* patches reduces due to dilution and the role of the *W* patch becomes more important due to transmission intensification and the dispersal of infection to other patches. Consequently, prevention may be achieved more efficiently by a strategy that also includes this patch.

If $R_0^2 > 1$ and infection is introduced into the population, an epidemic will occur, at least in the deterministic model. For epidemic management, we consider how the trajectory of this epidemic is affected by reducing some or all of the mosquito populations whenever a specified trigger incidence is reached in the entire human population. We consider several strategies based on applying the control to the mosquito populations in different subsets of patches. This model assumes that the control method is fumigation. In order to simulate the operational limitations encountered in the field a control decision is made every seven days, based on the incidence over the previous seven-day period. If incidence exceeds the trigger values, all of the mosquito populations included in the control strategy are reduced instantaneously to zero. Of course, such effective mosquito eradication is not possible in reality but, in our model, it provides a clear illustration of the potential impact of mosquito reduction measures on the epidemiological dynamics. The control is repeated whenever the seven-day incidence reaches the trigger level. As the mosquito population grows again, transmission returns and the epidemic builds again if the susceptible population is still sufficiently large. Nevertheless, transmission control stalls the epidemic momentum and can reduce the final epidemic size, as well as spreading the demands on the healthcare system.

**Fig. 5:**
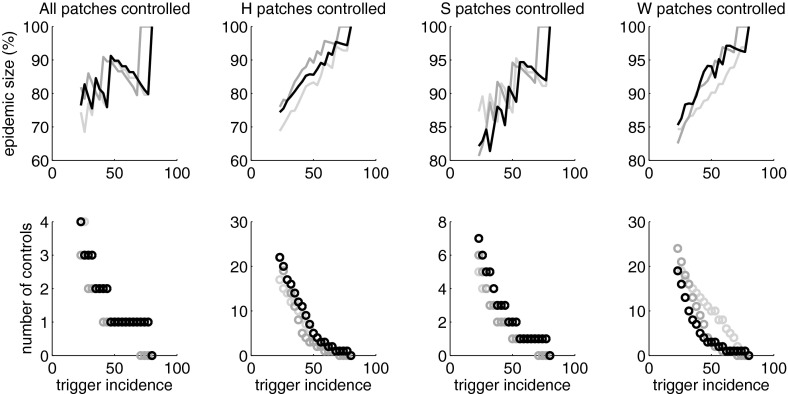
The epidemiological impact of applying mosquito control whenever the weekly incidence in the human population exceeds the trigger incidence. The *top row* shows the final epidemic size, in all patches and groups, relative to the final size of the corresponding uncontrolled epidemic. The *bottom row* shows the number of times the control is applied. The *first column* shows the result of applying the control to all five patches, the *second column* to patches $H_1$ and $H_2$ only, the *third column* to patches $S_1$ and $S_2$ only and the fourth column to patch *W* only. The *shade* indicates the proportion *q* of the total highly mobile (*HM*) and mixing commuter (*MC*) population that is in the *HM* group. *Light grey*: $q = 0.05$, *dark grey*: $q = 0.5$, *black*: $q = 0.95$. Parameters as in Table 1. Initial condition as in Fig. 3

Figure 5 shows the final epidemic size, relative to the final size of an uncontrolled epidemic, as a function of the trigger incidence. Applying the control to all the mosquito populations simultaneously can reduce the final epidemic size by up to 25 %. The reduction is largest when the trigger incidence is low. In this case, control is applied several times over the course of the epidemic. Higher trigger incidences tend to lead to less effective management, but the effect is not monotonic. The point in the epidemic at which the control is applied is critical because management is only achieved by reducing the epidemic momentum. It turns out that, if the trigger incidence is increased but the number of controls does not change, the epidemic management becomes more effective, i.e. the final epidemic size decreases. However, if the interplay of the control and the epidemic trajectory is such that increasing the trigger incidence causes one less control to be triggered over the course of the epidemic, there is a sharp drop in the effectiveness of the epidemic management; the final epidemic size is much larger. Some examples are shown in Supplementary Information, Figure S3. The *HM*–*MC* ratio has little impact on the effectiveness of this epidemic management strategy.

If the control is applied only to the *S* patches, it is triggered approximately twice as often, and is less effective, than when the control is applied to all patches. However, there is still a pattern of increased trigger incidences improving the effectiveness of management until the number of controls changes and the effectiveness suddenly drops again. If the control is applied only to the *H* or *W* patches, the pattern is different. Each control application is less effective at stalling the epidemic, largely because extensive transmission continues in the *S* patches. So these local controls are triggered frequently over the course of the epidemic. If the trigger incidence is increased, the number of controls and the management effectiveness decreases approximately linearly, at least until the number of controls becomes small. When the control is applied to the *W* patch only, the potential reduction in the final epidemic size is relatively modest because all the population groups that visit the *W* patch can also be infected elsewhere. However, when the control is applied to the *H* patches only, the impact on the final epidemic size can be commensurate with applying the control to all patches. The frequent controls virtually halt transmission in the *H* patches throughout the epidemic, effectively protecting the non-mobile population that is permanently resident in those patches. Simulations with the agent-based model show broadly consistent behaviour (Supplementary Information Figure S4) although the average of the stochastic trials does not show the sharp drops in the effectiveness of epidemic management observed in the deterministic model; this effect is smoothed away because the random variability in the epidemic trajectories leads to considerable variability in the number of controls that are applied.

## Discussion

A number of recent models have studied the role of human mobility in dengue virus transmission. These models often describe human movement as a commuting pattern between two places. However, field findings (Vazquez-Prokopec et al. 2009), and our group’s ongoing field observations using individual GPS tracking, have shown that day-to-day human movement in dengue endemic communities can involve multiple fixed destinations or random movement. In this paper, we have explored some of the epidemiological implications of a more complex commuting structure, and the basic mechanisms behind them. We modelled spatial structure as patches, each with a local mosquito population. These patches represent the broad locations in a city where people carry out their normal daily activities. We modelled population structure as subpopulations with mobility profiles broadly based on demographic characterisations. Some of these subpopulations have well-defined commuting patterns, others do not stray from their residential areas, others are highly mobile and spend short periods of time in each area of the city.

Our objective was to develop a model that has meaningful structure but remains simple enough for transparent analysis and interpretation. Of course, all mathematical models aim to capture the important features of the real-world system without unnecessary complexity, but the assessment of necessity is subjective and context dependent. Some modelling studies of dengue management strategies have employed detailed individual-based simulations developed, calibrated and validated using extensive field data. These simulation models have typically been used to assess the impact of a specific intervention, e.g. vaccination or mosquito control, in a specific population (Chao et al. 2012; Karl et al. 2014). The attention to detail in these models makes the broad correlations they reveal between different aspects of the system compelling. But the intricate complex of interactions between large numbers of components can render the mechanisms behind those correlations opaque and so limit the potential for generalisation. Consequently we did not attempt to simulate every aspect of the real world such as detailed individual movement patterns, temporal variation in behaviour driven by weekends or holidays (Danon et al. 2009; Martínez-Vega et al. 2012), variation in rainfall, temperature or breeding site availability (Otero and Solari 2010). The key drivers of the epidemiological dynamics in this study are dilution and connectivity. The relative simplicity of the model allows us to observe the complex mechanisms at work.

Our main parameter was the population size ratio between the highly mobile and mixing commuter groups. The highly mobile population moves frequently and is dispersed evenly over all patches. The mixing commuter populations are distributed between their residential patches and a common destination patch. Increasing the ratio transforms mixing commuters into highly mobile individuals, who disperse widely. This change intensifies transmission in the common destination patch, and to a small extent in the residential patch, because there are fewer people there at any given time. Conversely, it dilutes transmission in the other destination patches, those visited by the non-mixing commuter population, because there are more people there at any given time. Highly mobile individuals also disperse the infection risk, synchronising the epidemic trajectories in different population groups. The mosquito populations in patches in which transmission is intense because the vector–host ratio is high act as reservoirs and hubs of infection maintained and disseminated by the highly mobile group. In our model, the size of the mosquito population is the same in all patches. Consequently, the residential patches do not function as infection reservoirs because there are many people there at any given time and dilution is strong. Clearly the vector–host ratios could be adjusted non-uniformly to make the transmission intensity highest in the residential patches, or any other patch. A comprehensive analysis of the resultant plethora of combinations of host, mosquito and spatial heterogeneities would be laborious. However, we anticipate that non-uniform mosquito populations would change the way in which the epidemics in different population groups or patches are related, but not produce any fundamental new insights with regards the relation between dilution, connectivity and epidemic control.

In our model set-up, the transformation of individuals that commute to a common, centralised, destination to individuals that are highly mobile reduces the epidemic risk as quantified by the basic reproduction number. This reduction is primarily due to the dispersal of highly mobile individuals diluting transmission in the patches where it was previously most intense. The reduction in the epidemic risk saturates when the reduction in the mixing commuter population intensifies transmission in the common destination patch to the point where it compensates for the dilution in other patches. This suggests that, as a rule of thumb, in a moderately well-connected population the epidemic risk may be determined by the areas with the highest transmission intensity, i.e. the highest vector–host ratio. When an epidemic does occur, connectivity and dilution are also key factors governing its trajectory and composition. As a general rule, the epidemic grows more quickly in populations associated with patches where the vector–host ratio, and the transmission intensity, is highest. If the highly mobile population is small, and the connectivity between patches is rigidly structured, the sequence of epidemics in different population groups is governed by the extent of dilution in the patches they frequent. The spread of infection between populations associated with two patches may also be slowed by an intermediate patch in which dilution is more extensive. Conversely, a large highly mobile population synchronises the local epidemics associated with each patch. The size of the highly mobile population does not, however, have much impact on the final epidemic size as the infection reaches all patches and populations eventually.

Dengue epidemics may be stalled if transmission is controlled by insecticide fumigation. If the mosquito population quickly recovers to its former level, this type of control will not end the epidemic, but stalling can reduce the final epidemic size and limit health service saturation. For diseases such as dengue, this type of epidemic management may be a more realistic and immediate public health goal than eradication. The efficiency and effectiveness of the strategy depend on which mosquito populations are targeted, and the precise points in the epidemic at which the control is applied. Infrequent control is required if the targets include mosquito populations in patches visited by a considerable proportion of the population, but where dilution is not too extensive. In our case, these are the destination patches where commuters do not mix. Temporarily eradicating these mosquito populations reduces the epidemic momentum sharply and it may be some time before incidence increases sufficiently to trigger another control. The efficiency of these controls, in terms of reducing the final epidemic size, may be improved by waiting longer for the epidemic to grow more before applying them. However, this strategy is risky because waiting too long can result in sudden increase in final epidemic size. Frequent control is required if the targets do not include the mosquito populations in patches visited by a considerable proportion of the population, or where there is extensive dilution. In our case, these are the residential patches and the destination patch where commuters mix. Temporarily eradicating these mosquito populations once an epidemic is well underway has a limited impact on infection prevalence in any population group that comes into contact with the mosquito population elsewhere. If the groups involved are relatively large, transmission in these patches maintains overall epidemic momentum. Nevertheless, if there is a large population group that does not come into contact with the mosquito population elsewhere, eradicating the mosquito population in their local patch can have a significant impact on the final epidemic size. In our case, repeated controls to maintain very small mosquito populations in the residential patches proved to be an effective, although possibly not efficient, approach to managing the final epidemic size.

An obvious question to ask is, can movement restrictions prevent or control an epidemic? The insights gained from our model suggest that only the complete cessation of movement to a patch, or collection of patches, would be effective. A limited reduction of movement is worse than doing nothing. As before, the mechanism driving this observation is dilution. To see this, consider movement reduction modelled by converting a proportion of a commuter group to non-mobile individuals. The remainder of the commuter group continues to travel to and from their destination patch as usual. However, the reduction in commuter numbers increases the vector–host ratio in the destination patch, increasing the transmission intensity in the remaining commuter group, and any other group that visits that patch. Even if the entire commuter group is prevented from visiting their usual destination, if highly mobile individuals continue to visit that patch, it will be a transmission hotspot. These insights are derived from the deterministic model and will break down when population sizes become very small, but the general inference is instructive—locations should be quarantined, not people.

Intervention strategies to control dengue epidemics should, ideally, be informed by simulation models supported with high-quality field data. The simpler model we have presented here supports the development of those models by identifying key dynamical processes that influence how epidemic control is affected by human mobility, and providing quite general insights that may be helpful in the absence of detailed models. In particular, this modelling study helps to explain why the control of dengue outbreaks seems to be more difficult in some cities than in others. It is not just the number of mosquitoes that is important, but the fluctuating transmission landscape associated with spatial heterogeneity in the vector–host ratio and people’s daily movement patterns. In public health, one of the main parameters used for stratifying transmission risk areas is the estimated vector density, obtained by entomological indicators such as the Breteau index, house index, pupal index or positive ovitraps (Sanchez et al. 2006, 2010; Garelli et al. 2009; Pepin et al. 2013). The main objective of preventive measures is to keep these indicator values low. The importance of the dilution effect in our model suggests that these indicators should be extended to factor in the number of people spending at least part of their day in the area. It is clear that well-coordinated eradication of the mosquito population across the widest possible spatial area is the best way to reduce the risk of a dengue epidemic occurring, and to manage transmission if it does. However, logistic limitations can make this level of coverage difficult to achieve in the field. In this case, control targeted at specific areas can be a reasonably effective alternative.

### Electronic supplementary material

Below is the link to the electronic supplementary material.

Supplementary material 1 (pdf 629 KB)

Supplementary material 2 (txt 53 KB)

## Appendix: Complete System Equations

Non-mobile groups ($NM_i$)

12 $$\begin{aligned} \frac{\mathrm{d}S_{NM_i}^{H_i}}{\mathrm{d}t}= & {} -\beta I_V^{H_i}\frac{S_{NM_i}^{H_i}}{N_\varSigma ^{H_i}} \end{aligned}$$

13 $$\begin{aligned} \frac{\mathrm{d}E_{NM_i}^{H_i}}{\mathrm{d}t}= & {} \beta I_V^{H_i}\frac{S_{NM_i}^{H_i}}{N_\varSigma ^{H_i}} - \varepsilon E_{NM_i}^{H_i} \end{aligned}$$

14 $$\begin{aligned} \frac{\mathrm{d}I_{NM_i}^{H_i}}{\mathrm{d}t}= & {} \varepsilon E_{NM_i}^{H_i} - \gamma I_{NM_i}^{H_i} \end{aligned}$$

15 $$\begin{aligned} \frac{\mathrm{d}R_{NM_i}^{H_i}}{\mathrm{d}t}= & {} \gamma I_{NM_i}^{H_i} \end{aligned}$$

where $$i = 1$$ or 2, $$N_\varSigma ^{H_i}= N_{NM_i}^{H_i}+N_{NC_i}^{H_i} +N_{MC_i}^{H_i} + N_{HM}^{H_i}$$.

Non-mixing commuter groups ($NC_i$)

16 $$\begin{aligned} \frac{\mathrm{d}S_{NC_i}^{H_i}}{\mathrm{d}t}= & {} -\beta I_V^{H_i}\frac{S_{NC_i}^{H_i}}{N_\varSigma ^{H_i}} - \tau _{NC}S_{NC_i}^{H_i} + \rho _{NC}S_{NC_i}^{S_i} \end{aligned}$$

17 $$\begin{aligned} \frac{{\mathrm{d}E}_{NC_i}^{H_i}}{\mathrm{d}t}= & {} \beta I_V^{H_i}\frac{S_{NC_i}^{H_i}}{N_\varSigma ^{H_i}} - \varepsilon E_{NC_i}^{H_i} - \tau _{NC}E_{NC_i}^{H_i} + \rho _{NC}E_{NC_i}^{S_i}\end{aligned}$$

18 $$\begin{aligned} \frac{{\mathrm{d}I}_{NC_i}^{H_i}}{\mathrm{d}t}= & {} \varepsilon E_{NC_i}^{H_i} - \gamma I_{NC_i}^{H_i} - \tau _{NC}I_{NC_i}^{H_i} + \rho _{NC}I_{NC_i}^{S_i}\end{aligned}$$

19 $$\begin{aligned} \frac{{\mathrm{d}R}_{NC_i}^{H_i}}{\mathrm{d}t}= & {} \gamma I_{NC_i}^{H_i} - \tau _{NC}R_{NC_i}^{H_i} + \rho _{NC}R_{NC_i}^{S_i} \end{aligned}$$

20 $$\begin{aligned} \frac{{\mathrm{d}S}_{NC_i}^{S_i}}{\mathrm{d}t}= & {} -\beta I_V^{S_i}\frac{S_{NC_i}^{S_i}}{N_\varSigma ^{S_i}} - \rho _{NC}S_{NC_i}^{S_i} + \tau _{NC}S_{NC_i}^{H_i} \end{aligned}$$

21 $$\begin{aligned} \frac{{\mathrm{d}E}_{NC_i}^{S_i}}{\mathrm{d}t}= & {} \beta I_V^{S_i}\frac{S_{NC_i}^{S_i}}{N_\varSigma ^{S_i}} - \varepsilon E_{NC_i}^{S_i} - \rho _{NC}E_{NC_i}^{S_i} + \tau _{NC}E_{NC_i}^{H_i}\end{aligned}$$

22 $$\begin{aligned} \frac{{\mathrm{d}I}_{NC_i}^{S_i}}{\mathrm{d}t}= & {} \varepsilon E_{NC_i}^{S_i} - \gamma I_{NC_i}^{S_i} - \rho _{NC}I_{NC_i}^{S_i} + \tau _{NC}I_{NC_i}^{H_i}\end{aligned}$$

23 $$\begin{aligned} \frac{{\mathrm{d}R}_{NC_i}^{S_i}}{\mathrm{d}t}= & {} \gamma I_{NC_i}^{S_i} - \rho _{NC}R_{NC_i}^{S_i} + \tau _{NC}R_{NC_i}^{H_i} \end{aligned}$$

where $i = 1$ or 2, $N_\varSigma ^{H_i} = N_{NM_i}^{H_i} + N_{NC_i}^{H_i} + N_{MC_i}^{H_i} +N_{HM}^{H_i}$ and $N_\varSigma ^{S_i}=N_{NC_i}^{S_i} + N_{HM}^{S_i}$.

Mixing commuter groups ($MC_i$)

24 $$\begin{aligned} \frac{{\mathrm{d}S}_{MC_i}^{H_i}}{\mathrm{d}t}= & {} -\beta I_V^{H_i}\frac{S_{MC_i}^{H_i}}{N_\varSigma ^{H_i}} - \tau _{MC}S_{MC_i}^{H_i} + \rho _{MC}S_{MC_i}^{W} \end{aligned}$$

25 $$\begin{aligned} \frac{{\mathrm{d}E}_{MC_i}^{H_i}}{\mathrm{d}t}= & {} \beta I_V^{H_i}\frac{S_{MC_i}^{H_i}}{N_\varSigma ^{H_i}} - \varepsilon E_{MC_i}^{H_i} - \tau _{MC}E_{MC_i}^{H_i} + \rho _{MC}E_{MC_i}^{W} \end{aligned}$$

26 $$\begin{aligned} \frac{{\mathrm{d}I}_{MC_i}^{H_i}}{\mathrm{d}t}= & {} \varepsilon E_{MC_i}^{H_i} - \gamma I_{MC_i}^{H_i} - \tau _{MC}I_{MC_i}^{H_i} + \rho _{MC}I_{MC_i}^{W} \end{aligned}$$

27 $$\begin{aligned} \frac{{\mathrm{d}R}_{MC_i}^{H_i}}{\mathrm{d}t}= & {} \gamma I_{MC_i}^{H_i} - \tau _{MC}R_{MC_i}^{H_i} + \rho _{MC}R_{MC_i}^{W} \end{aligned}$$

28 $$\begin{aligned} \frac{{\mathrm{d}S}_{MC_i}^{W}}{\mathrm{d}t}= & {} -\beta I_V^{W}\frac{S_{MC_i}^{W}}{N_\varSigma ^{W}} - \rho _{MC}S_{MC_i}^{W} + \tau _{MC}S_{MC_i}^{H_i} \end{aligned}$$

29 $$\begin{aligned} \frac{{\mathrm{d}E}_{MC_i}^{W}}{\mathrm{d}t}= & {} \beta I_V^{W}\frac{S_{MC_i}^{W}}{N_\varSigma ^{W}} - \varepsilon E_{MC_i}^{W} - \rho _{MC}E_{MC_i}^{W} + \tau _{MC}E_{MC_i}^{H_i} \end{aligned}$$

30 $$\begin{aligned} \frac{{\mathrm{d}I}_{MC_i}^{W}}{\mathrm{d}t}= & {} \varepsilon E_{MC_i}^{W} - \gamma I_{MC_i}^{W} - \rho _{MC}I_{MC_i}^{W} + \tau _{MC}I_{MC_i}^{H_i} \end{aligned}$$

31 $$\begin{aligned} \frac{{\mathrm{d}R}_{MC_i}^{W}}{\mathrm{d}t}= & {} \gamma I_{MC_i}^{W} - \rho _{MC}R_{MC_i}^{W} + \tau _{MC}R_{MC_i}^{H_i} \end{aligned}$$

where $i = 1$ or 2, $N_\varSigma ^{H_i} = N_{NM_i}^{H_i} + N_{NC_i}^{H_i} +N_{MC_i}^{H_i} +N_{HM}^{H_i}$ and $N_\varSigma ^W = N_{MC_1}^W+ N_{MC_2}^W + N_{HM}^{W}$.

Highly mobile group (*HM*)

32 $$\begin{aligned} \frac{{\mathrm{d}S}_{HM}^Y}{\mathrm{d}t}= & {} -\beta I_V^Y\frac{S_{HM}^Y}{N_\varSigma ^Y} - \rho _{HM}S_{HM}^Y + \frac{\rho _{HM}}{4}S_{HM}^{\varSigma \setminus Y} \end{aligned}$$

33 $$\begin{aligned} \frac{{\mathrm{d}E}_{HM}^{Y}}{\mathrm{d}t}= & {} \beta I_V^{Y}\frac{S_{HM}^{Y}}{N_\varSigma ^{Y}} - \varepsilon E_{HM}^Y - \rho _{HM}E_{HM}^{Y} + \frac{\rho _{HM}}{4}E_{HM}^{\varSigma \setminus Y} \end{aligned}$$

34 $$\begin{aligned} \frac{{\mathrm{d}I}_{HM}^{Y}}{\mathrm{d}t}= & {} \varepsilon E_{HM}^Y -\gamma I_{HM}^Y - \rho _{HM}I_{HM}^{Y} + \frac{\rho _{HM}}{4}I_{HM}^{\varSigma \setminus Y} \end{aligned}$$

35 $$\begin{aligned} \frac{{\mathrm{d}R}_{HM}^{Y}}{\mathrm{d}t}= & {} \gamma I_{HM}^Y - \rho _{HM}R_{HM}^{Y} + \frac{\rho _{HM}}{4}R_{HM}^{\varSigma \setminus Y} \end{aligned}$$

where $Y = H_1, H_2, S_1, S_2$ or $W, N_\varSigma ^Y = N_{NM_1}^Y+N_{NM_2}^Y+N_{NC_1}^Y + N_{NC_2}^Y+N_{MC_1}^Y + N_{MC_2}^Y+ N_{HM}^Y$ and $Z_{HM}^{\varSigma \setminus Y}=Z_{HM}^{H_1}+Z_{HM}^{H_2}+Z_{HM}^{S_1} +Z_{HM}^{S_2}+Z_{HM}^W-Z_{HM}^Y$ for all infection states *Z*.

Mosquitoes

36 $$\begin{aligned} \frac{{\mathrm{d}S}_{V}^{Y}}{\mathrm{d}t}= & {} \frac{\kappa N}{5}\mu - \beta S_V^Y\frac{I_\varSigma ^Y}{N_\varSigma ^Y} - \mu S_V^Y \end{aligned}$$

37 $$\begin{aligned} \frac{{\mathrm{d}E}_{V}^{Y}}{\mathrm{d}t}= & {} \beta S_V^Y\frac{I_\varSigma ^Y}{N_\varSigma ^Y} - \varepsilon _V E_V^Y - \mu E_V^Y \end{aligned}$$

38 $$\begin{aligned} \frac{{\mathrm{d}I}_{V}^{Y}}{\mathrm{d}t}= & {} \varepsilon _V E_V^Y - \mu I_V^Y \end{aligned}$$

where $$Y = H_1, H_2, S_1, S_2$$ or $$W, N_\varSigma ^Y = N_{NM_1}^Y+N_{NM_2}^Y+N_{NC_1}^Y+N_{NC_2}^Y +N_{MC_1}^Y +N_{MC_2}^Y+N_{HM}^Y$$.
