# Supplementary material for: Day-to-Day Population Movement and the Management of Dengue Epidemics
Source: Bull Math Biol. 2016 Oct 4;78(10):2011–33. doi: 10.1007/s11538-016-0209-6 (PMC5069346; doi:10.1007/s11538-016-0209-6)
Supplement: Supplementary file 1 — Supplementary material 1 (pdf 629 KB) [file 11538_2016_209_MOESM1_ESM.pdf]

# Supplementary Information

## Day-to-day population movement and the management of dengue epidemics

Falcón-Lezama, Martínez-Vega, Kuri-Morales, Ramos-Castañeda and Adams

## 1. Stochastic model

The ordinary differential equation system was re-formulated as a discrete-time stochastic agent-based simulation using Netlogo 5.0.4. The agent-based model was constructed to be as similar as possible to the ODE system. Individual people were represented as agents characterised by their movement behaviour (non-mobile, mixing commuter, non-mixing commuter, highly mobile), habitual locations, and infection state (S, E, I, R). Netlogo 5 does not have a dedicated framework for network dynamics. So locations were also represented as 'agents' characterised by the number of mosquitoes in that location in each infections state (S, E, I). Spatial structure was then achieved by assigning each person agents to be in contact with a unique location agent. The system was simulated with a two-hour time step $\Delta t$. Whenever a person agent was assigned to, or arrived in, a location, their sojourn time was calculated and after this time had elapsed they were moved again. Sojourn times were taken to be exponentially distributed with means given by the relevant $\rho$ and $\tau$ parameters. The mosquito population of each patch was modelled en masse, not as agents. In each time step, the number of mosquito births in a patch was taken to be Poisson distributed with rate $\frac{\kappa N}{5}\mu\Delta t$; the number of deaths in the each mosquito population $Z_V^Y$ was taken to be binomially distributed with $p = \mu\Delta t$; agents in state $S_X^Y$ became infected with probability $1 - \exp\left(-\beta \frac{I_V^Y}{N_\Sigma^Y}\Delta t\right)$ where $N_\Sigma^Y$ was the total number of agents in patch $Y$; the number of infections in the $S_V^Y$ mosquito population was taken to be binomially distributed with $p = \beta\frac{I_\Sigma^Y}{N_\Sigma^Y}\Delta t$; the number of mosquitoes in the $E_V^Y$ population that progressed to the infectious state was taken to be binomially distributed with $p = \epsilon_V \Delta t$. For agents, the durations of the exposed and infectious states were taken to be exponentially distributed with means $1/\mu$ and $1/\gamma$ respectively. The full Netlogo code is included with this Supplementary Material as a separate file.

## 2. Supplementary figures

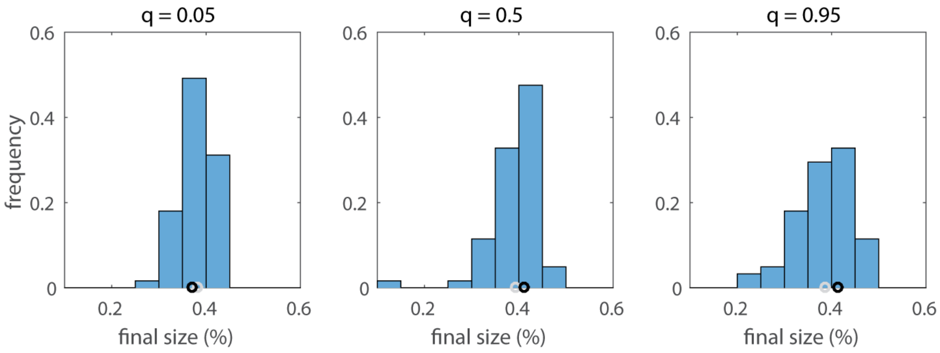


Figure S1: Distributions of final epidemic size under the stochastic model depending on the proportion $q$ of the total highly mobile ($HM$) and mixing commuter ($MC$) population in the $HM$ group. Each graph shows the frequency of trials that resulted in the given proportion of the population being infected (and recovering) by the end of the epidemic ($\sum_X R_X(1000)/N$). There are 61 independent trials for each value of $q$. The pale circles are the means of stochastic realisations. The black circles are the output of the deterministic ODE model. Parameters as in Table 1. Initially all individuals were susceptible except five which were set to be exposed. For the stochastic model these individuals were chosen at random. For the deterministic model they were distributed evenly over all 15 exposed classes of the human population i.e. $S_X^Y(0) = N_X^Y(0) - 5/15, E_X^Y(0) = 5/15$ for all 15 valid combinations of group $X$ and patch $Y$. Stochastic realisations were required to result in a final epidemic size of at least 10 % of the population. Realisations for which this did not occur were repeated.

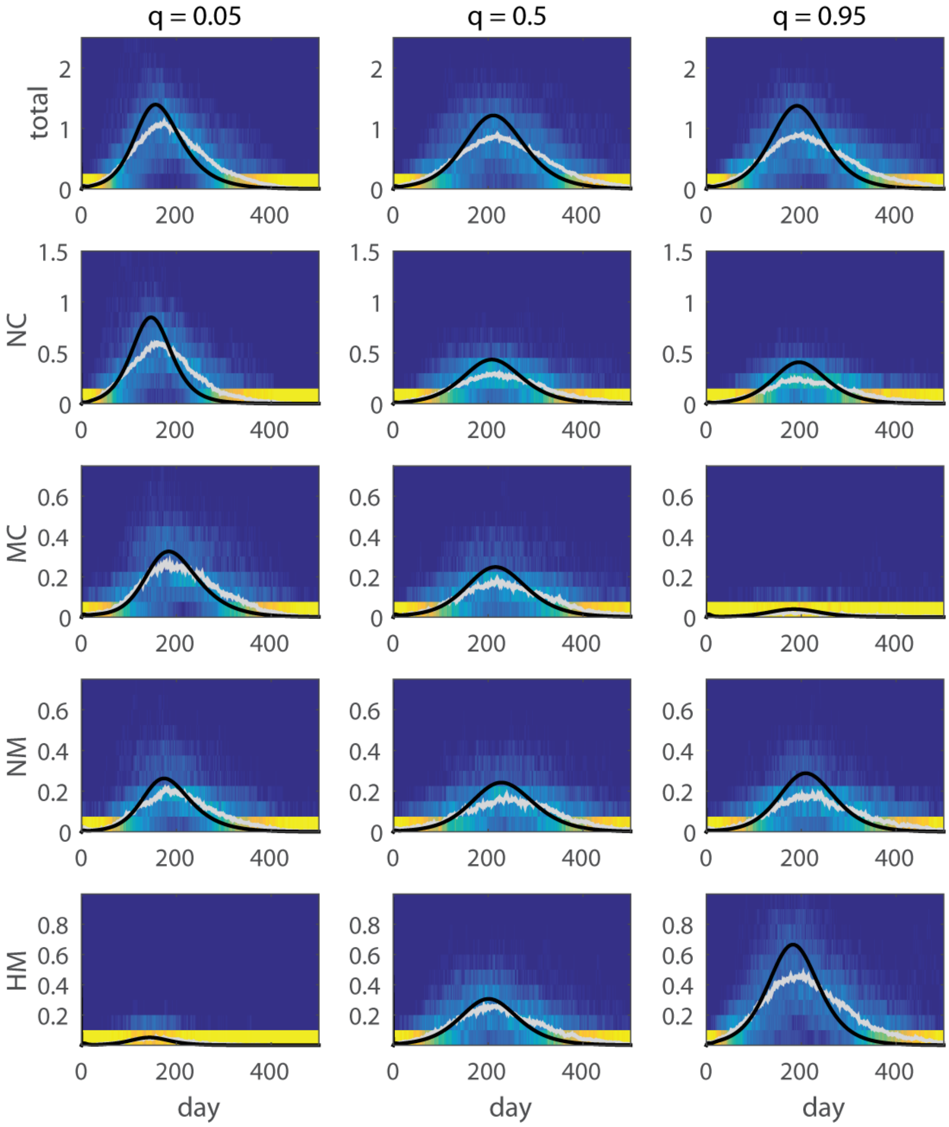


Figure S2: Stochastic and deterministic realisations of the epidemic trajectory and its constituents depending on the proportion $q$ of the total highly mobile ($HM$) and mixing commuter ($MC$) population in the $HM$ group. Each graph shows percentage prevalence over time as a proportion of the total population for specific groups ($I_X(t)/N \times 100$, for $X = NM, NC, MC, HM$) and the total population $\sum_X I_X(t)/N \times 100$. Rows correspond to different population groups. Columns correspond to different values of $q$. The pale grey lines in each column are the means of 61 stochastic realisations of the system. The background colours indicate the densities of these realisations (vertically at each time point) yellow is highest, dark blue is lowest. The black lines are the output of the deterministic ODE model. All model output is the same as used to construct Figure S1.

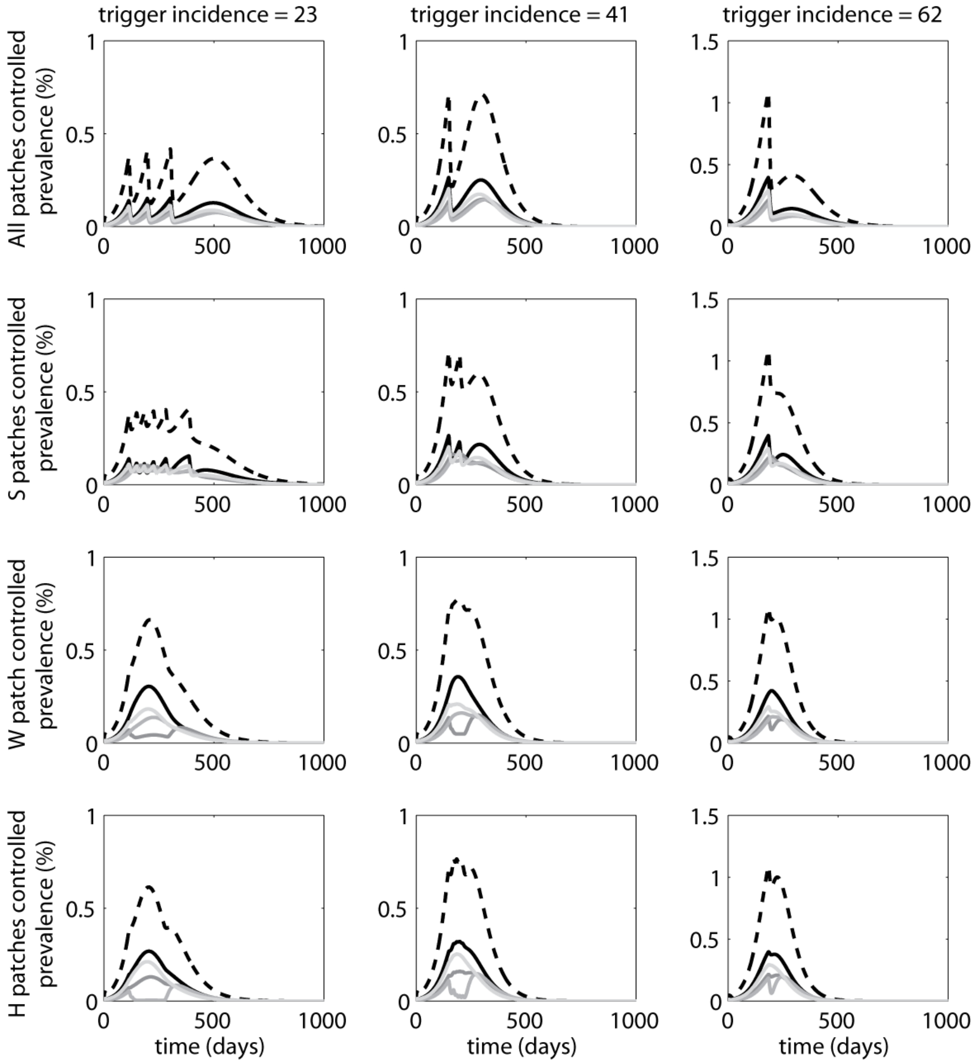


Figure S3: Epidemic trajectories from the deterministic model under different control strategies. Each panel shows the total infection prevalence as a percentage of the total population $\sum_X I_X(t)/N \times 100$, broken down into demographic groups. Dashes: total prevalence. Shades indicate the contribution to the total prevalence from different groups. Black: non-mixing commuters ($NC$). Dark grey: mixing commuters ($MC$). Grey: non-mobile ($NM$). Light grey: highly mobile ($HM$). Each column corresponds to a different weekly incidence at which the control is triggered. Each row corresponds to a control strategy in which a different subset of the mosquito populations are reduced to 0 each time a control is triggered. The proportion of the total highly mobile ($HM$) and mobile commuter ($MC$) population that is in the $HM$ group $q = 0.5$. Other parameters as in Table 1. Initial conditions as described for Figure S1.

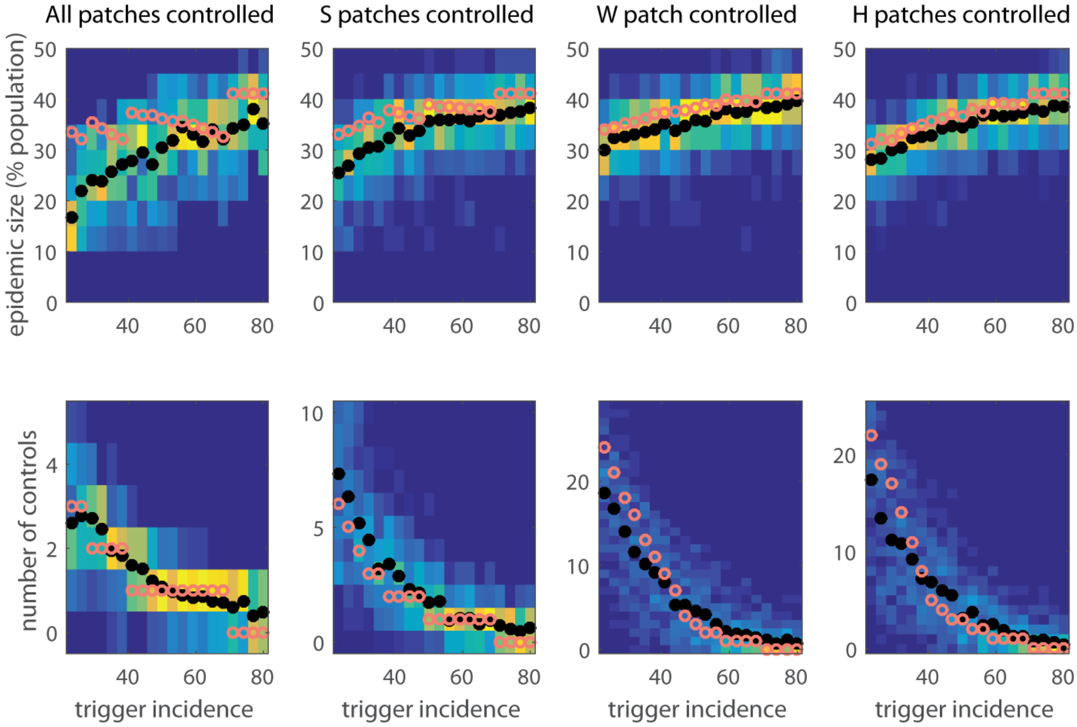


Figure S4: Stochastic realisations of the epidemiological impact of applying mosquito control whenever the weekly incidence in the human population exceeds the trigger value. The top row shows the final epidemic size as a percentage of the total population $(\sum_X R_X(1000)/N \times 100)$. Note that this is different to Figure 5 in the main text, where the final epidemic size is shown relative to the final size of the uncontrolled epidemic. The bottom row shows the number of times the control is applied. The first column shows the result of applying the control to all five patches, the second column to patches $S_1$ and $S_2$ only, the third column to patch $W$ only and the fourth column to patches $H_1$ and $H_2$ only. The black circles in each column are the means of 50 stochastic realisations for each trigger incidence. The background colours indicate the densities of these outcomes (vertically at each trigger value) yellow is highest, dark blue is lowest. The red circles are the output of the corresponding deterministic ODE model. The proportion of the total highly mobile $(HM)$ and mixing commuter $(MC)$ population that is in the $HM$ group $q = 0.5$. Other parameters as in Table 1. Initial conditions as described for Figure S1. Stochastic realisations were required to result in a final epidemic size of at least 10 % of the population. Realisations for which this did not occur were repeated.
